# C5a Blockade Increases Regulatory T Cell Numbers and Protects Against Microvascular Loss and Epithelial Damage in Mouse Airway Allografts

**DOI:** 10.3389/fimmu.2018.01010

**Published:** 2018-05-24

**Authors:** Mohammad Afzal Khan, Fatimah Alanazi, Hala Abdalrahman Ahmed, Axel Vater, Abdullah Mohammed Assiri, Dieter Clemens Broering

**Affiliations:** ^1^Comparative Medicine Department, King Faisal Specialist Hospital and Research Centre, Riyadh, Saudi Arabia; ^2^Organ Transplant Centre, King Faisal Specialist Hospital and Research Centre, Riyadh, Saudi Arabia; ^3^Aptarion Biotech AG, Berlin, Germany; ^4^College of Medicine, AlFaisal University, Riyadh, Saudi Arabia; ^5^Institute for Research and Medical Consultations, Imam Abdulrahman Bin Faisal University, Dammam, Saudi Arabia

**Keywords:** C5a blocking, microvascular reestablishment, Treg induction, immunosuppression, airway allografts

## Abstract

Microvascular injury during acute rejection has been associated with massive infiltration of CD4^+^ T effector cells, and the formation of complement products (C3a and C5a). Regulatory T cells (Tregs) are potent immunosuppressors of the adaptive immune system and have proven sufficient to rescue microvascular impairments. Targeting C5a has been linked with improved microvascular recovery, but its effects on the Treg and T effector balance is less well known. Here, we demonstrate the impact of C5a blockade on Treg induction and microvascular restoration in rejecting mouse airway allografts. BALB/c→C57BL/6 allografts were treated with a C5a-neutralizing l-aptamer (10 mg/kg, i.p. at d0 and every second day thereafter), and allografts were serially monitored for Treg infiltration, tissue oxygenation (tpO_2_), microvascular blood flow, and functional microvasculature between donor and recipients during allograft rejection. We demonstrated that C5a blocking significantly leads to enhanced presence of Tregs in the allograft, reinstates donor–recipient functional microvasculature, improves tpO_2_, microvascular blood flow, and epithelial repair, followed by an upregulation of IL-5, TGF-β, IL-10 vascular endothelial growth factor, and ANGPT1 gene expression, while it maintained a healthy epithelium and prevented subepithelial collagen deposition at d28 posttransplantation. Together, these data indicate that inhibition of C5a signaling has potential to preserve microvasculature and rescue allograft from a sustained hypoxic/ischemic phase, limits airway tissue remodeling through the induction of Treg-mediated immune tolerance. These findings may be useful in designing anti-C5a therapy in combination with existing immunosuppressive regimens to rescue tissue/organ rejection.

## Introduction

Promoting microvascular integrity supports transplant health and may avoid the onset of chronic rejection (CR) of transplanted lungs. Allograft microvascular injuries have been associated with massive infiltration of CD4^+^ T effector cells and antibody-mediated complement activation, which promotes obliteration of microvasculature, airway remodeling, and ultimately CR of transplanted lungs (1–5). During the early phase of inflammation, complement pathways release active complement fragments, C3a, C4a, and C5a. These are potent anaphylatoxins that alter vascular permeability and airway remodeling (6–9). In normal circumstances, the C3 split product C3b plays a central role in generating C5 convertase to cleave C5 into the active components C5a and C5b. Compared to the other C3 split product, C3a, C5a is the more potent chemotactic agent, and is recognized by C5aR on a variety of lymphocytes including regulatory T cells (Tregs) during inflammation, and further binding of C5a during the inflammatory phase results in shifting of the T cell response to a Th1-type, promoting effector T cell activation (10) and limiting natural regulatory T cell (nTreg) regulatory activities (11). Of note, C5a, being the most potent anaphylatoxin, is considered the biologically most lethal fragment released during complement activation, and is therefore, regarded as a key target for complement modulation (12, 13). Although C5 cleavage generates C5a as well as C5b which forms together with C6, C7, C8 and C9 the lytic terminal Membrane Attack Complex, the clinical outcomes of C5 deficiency do not differ markedly from those of other terminal component deficiencies (e.g., C6, C7, C8, and C9) suggesting that the absence of C5a does not contribute significantly to the clinical aftermath in C5-deficient patients. Therefore, the selective blockade of C5a promises to be the optimal leverage, so that the normal up- and downstream disease-preventing functions of complement remain intact, and only the harmful activated fragment, the pro-inflammatory anaphylatoxin, is blocked.

CD4^+^CD25^+^FOXP3^+^ Tregs were first reported to be crucial for the maintenance of self-tolerance, for immune homeostasis and for protection from autoimmune diseases (14). Forkhead box P3 (FOXP3) is an intracellular marker, which is specifically expressed in regulatory CD4^+^CD25^+^ in the course of differentiation from naïve CD4^+^ T cells. Tregs play a central role in establishing immunological tolerance and suppress autoimmunity (15, 16). A number of preclinical and clinical studies highlighted the therapeutic potential of these cells in organ transplantation, but pathological or beneficial effects of Tregs on rejecting allografts are still not very clear (15, 17–23).

Regulatory T cells modulate immune balance and self-tolerance through the generation of thymus-derived Tregs and peripheral Treg infiltration, and in the last couple of years, the immunosuppressive property of Treg has been utilized to rescue transplants during rejection (24). CD4^+^FOXP3^+^ peripheral Tregs play a fundamental role in maintaining immunotolerance against mucosal injury and pathogenic alloimmunity. In addition, they facilitate tolerance induction in murine models of solid organ transplantation (3–6). In contrast to thymus-derived Tregs, peripheral Tregs are liable to inflammation-induced reversal into effector T cells (8), thereby limiting their therapeutic potential in autoimmune diseases. Thymus-derived Tregs are critical regulators of self-tolerance (25) and protect against pathogenic alloimmunity (26), and have been shown to facilitate tolerance induction in murine models of solid organ (24, 27) and hematopoietic cell transplantation (28). In general, Tregs are known to be crucial in the maintenance of peripheral immune tolerance, and are the key modulators of the immune reaction during allograft maintenance in a number of transplant models including kidney, cardiac, and post-ischemic neovascularization in hind limb ischemia (29–36).

Previously, we reported that Treg-mediated immunotherapy has potential to preserve the microvasculature of transplanted tracheae and thereby to rescue the allografts from a sustained hypoxic/ischemic phase, which limits airway tissue remodeling (23, 37). Our findings demonstrated that Treg reconstitution favored reestablishment of donor–recipient microvasculature as compared to rejecting allografts on all time points studied. Furthermore, this study highlighted an interesting inverse correlation between the duration of microvascular loss (hypoxia/ischemia) and the presence of Tregs in blood and tissue during rejection episodes, which critically affected inflammation-induced epithelial loss, and subepithelial fibrosis.

It has been shown previously that C3a and C5a both activate T cells and antigen-presenting cells (APC), and that signaling of C3aR/C5aR on T cells and APCs influences T cell differentiation, expansion and survival of CD4^+^ T helper cells, which suggests that complement deficiency or blockade can potentially attenuate T cell-mediated autoimmunity (T regulatory and T effector cell balance) and thus delay allograft rejection (38). Thus, blocking and deficiency of both C5a and C5aR have been associated with improved allograft survival (39, 40). In addition, expression of and signaling through both C3aR and C5aR on nTregs has been reported to inhibit Treg function (3, 41), while pharmacological blockade of C5a has been associated with increased frequency of Tregs in a mouse GvHD model and with increased microvascular and epithelial repair in murine airway allografts (7, 24, 42). These findings prompted us to investigate a possible relationship between the complement cascade and T regulatory cells, which when combined may further amplify the regulatory mechanism of tissue recipients. The current study pursued to uncover the novel therapeutic benefits of AON-D21, an l-aptamer binding to C5a and to the C5a moiety on C5 prior to cleavage into C5a and C5b. AON-D21 therapy led to Treg induction, improvement in microvascular blood flow, and associated tissue repair in an experimental mouse model of orthotopic airway allograft (43), which resembles the airway fibrosis seen in clinical lung transplantation (1, 44). Therefore, the key objective was to demonstrate if therapeutic administration of the C5a inhibitor AON-D21 may facilitate microvascular blood flow of allografts through Treg induction and thus promote tissue oxygenation (tpO_2_), and limit fibrosis during rejection. This novel therapeutic strategy of targeting the C5a C5aR-axis during acute rejection episodes may be an effective therapeutic strategy in combination with other immunosuppressive regimens for preventing the development of CR.

## Materials and Methods

### Mice

All mice utilized in this study were originally purchased from the Jackson laboratory (JAX, USA), and later maintained as an original colony in our animal research facility at King Faisal specialist Hospital and Research Center (KFSH&RC), Riyadh, Kingdom of Saudi Arabia.

### C57BL/6

C57BL/6 (B6.H-2^b^) mice were utilized as recipients of all grafts and as tracheal donors in syngrafts as described below (Table 1).

**Table 1:** Experimental groups.

Donor	Recipient	Treatment	Group purpose	Assessments (days)
C57BL/6	C57BL/6	Vehicle	Vehicle-treated syngeneic control	4, 8–14, 20, 28
BALB/c	C57BL/6	Vehicle	Vehicle-treated allogeneic control	4, 8–14, 20, 28
BALB/c	C57BL/6	AON-D21	C5a inhibition	4, 8–14, 20, 28
BALB/c	C57BL/6	Anti-CD25 + AON-D21	Regulatory T cell depletion and C5a inhibition	8

*Sample size (*n*) = 4–6 transplants/time point/experiment (repeats of three experiments)*.

### BALB/c

BALB/c (H-2^d^) mice were routinely used as allogeneic tracheal donors for the B6 strain in the experimental allogeneic orthotopic trachea transplants. Based on our previously published findings (7, 45, 46), we selected days 4, 8, 9, 10–14, 20, and 28 for studying microvascular flow and tissue hypoxia in experimental groups (Table 1).

### Orthotopic Tracheal Transplant (OTT) Model

The mouse model of OTT is a well-established experimental model of alloimmune airway transplant rejection (7, 24, 43, 45, 46). Currently, there are no models which consistently produce and replicate fibrotic remodeling as seen in transplant recipients during the progressive development of CR in terminal bronchioles (47). However, the OTT model is a model of large airway transplantation and successfully replicates the development of fibrotic remodeling that develops to lymphocytic bronchitis, which is a key phase of large airway inflammation that can precede CR (1, 2, 44). Anatomically, orthotopic grafts remain exposed to air circulation, which provides a unique respiratory interface between donor–recipients microvascular blood flow after transplantation to favor natural physiological conditions. In addition, the orthotopic position of the graft further provides the preservation of the APC and lymphocyte trafficking pathways, which is very critical for the investigation of ongoing airway alloimmune responses during rejection (43). Based on previous studies, this is the most suitable model to examine donor–recipient microvascular connections, the real-time monitoring of blood flow and graft oxygenation during rejection episodes.

### Surgical Procedure

King Faisal specialist Hospital and Research Center Animal Care and Use Committee approved the experimental protocol adopted in this study. C57BL/6 (B6.H-2^b^) were transplanted under sterile conditions with tracheas from B6 mice (syngeneic tracheal graft) and MHC-mismatched BALB/c (H-2^d^) mice (allogeneic tracheal graft). 4–6 ring long tracheal segments from CO_2_ euthanized donor mice were dissected out and used as a grafts. C57BL/6 recipient mice were anesthetized with ketamine (100 mg/kg) and xylazine (20 mg/kg), and a short incision was made in the middle neck region, which allowed division of strap muscles and visualization of the entire laryngotracheal complex. Finally, donor and recipient tracheas were connected together with 10-0 nylon suture and the overlying skin was closed with 5-0 silk suture (43, 48). All transplanted animals were given post-operative medication Carprofen (dose 5 mg/kg SC) and Zolecin (dose 100 mg/kg SC), and were regularly monitored for any respiratory distress-stridor in the first 24 h after surgery, and in case of any respiratory distress, the recipients were immediately euthanized by CO_2_.

### Depletion of CD25^+^ Tregs

C57BL/6 mice were given i.p. injections of anti-mouse CD25 (clone PC61) from BioXCell, USA (250 μg/day). To get maximum Treg depletion, all mice were treated for at least 7 days prior to the day of transplantation (24, 27, 49). Treg depletion was confirmed by flow cytometry using CD25 staining on CD4^+^FOXP3^+^ T cells.

### AON-D21 and Blocking of C5a

AON-D21 is a PEGylated C5a-binding mixed RNA/DNA l-configured aptamer (50). The molecular weight of the oligonucleotide portion is 13,167 Da. PEGylation with Y-shaped 40 kDa methoxy polyethylene glycol is done in order to increase the plasma half-life, mainly by reducing glomerular filtration. Due to its un-natural (mirror image) l-configuration, the aptamer AON-D21 is resistant to nuclease attack in blood (51). AON-D21 originates from the l-aptamer NOX-D19, but has an improved affinity and may be easier to produce because it only consists of 40 nucleotides, whereas NOX-D19 consisted of 44 nucleotides (7). To study the effects of the C5a neutralization, BALB/c (H-2^d^) → C57BL/6 (H-2^b^) allografts were given 10 mg/kg, of AON-D21 or vehicle (5% glucose) by i.p. injections on d0 and every second day thereafter.

### Flow Cytometric Analysis

Blood was collected at selected time points posttransplantation through retro-orbital bleeding and collected in BD vacutainers. Briefly, lymphocytes were isolated by the Histopaque gradient separation method (52). After centrifugation at 400 × *g* for 30 min, the buffy coat of lymphocytes was aspirated, and specific Treg markers (CD4, CD25, and FOXP3) were stained with fluorescently labeled rat anti-mouse antibodies, i.e., APC-CD4^+^ (Clone RM4-5 RUO), PE-Cy7 CD25^+^ (Clone PC 61 RUO), and PE-conjugated FOXP3^+^ (Clone MF23 RUO), respectively, as recommended by BD Pharmingen FOXP3 fixation and permeabilization assay, which specifically flow sort the CD4^+^CD25^+^FOXP3^+^ Treg subpopulation from mixed lymphocytes in systemic circulation. All were referenced to isotype control mouse IgG1-fluorescein isothiocyanate (FITC) (ab91356, abcam) to determine the background due to nonspecific antibody binding, and T cells were confirmed with CD4 and CD3 coexpression. Data were recorded at the flow rate of 14 μl/min and a minimum of 500,000 events were collected, and further analyzed through BD Accuri integrated software version C6.

### Immunofluorescence Staining

All rejecting and anti-C5a treated allograft specimens were further examined by immunohistochemical staining for CD4^+^FOXP3^+^ Tregs in grafts. Briefly, tracheal transplants were harvested and processed in Tissue-Tek O.C.T. medium (Sakura Finetek, Japan). A cryostat (HM550; Microm) was utilized to cut 5 µm sections of the tracheal grafts and the sections were placed on superfrost/plus slides (Fisher Scientific). After fixation in methanol/acetone (1:1) for 10 min at −20°C, the slides were washed with phosphate buffered saline (PBS). Next, graft sections were incubated with 10% donkey serum for 30 min and then incubated for 1 h with either rat anti-mouse CD4 (BD biosciences, USA) or rabbit anti-mouse FOXP3 (abcam, USA) primary antibodies. The slides were then washed with PBS, and sections were further incubated for 1 h with Cy3 donkey anti-rat (Jackson Immuno research, USA) and Alexa 647 donkey anti-rabbit (Jackson Immuno research, USA) secondary antibodies. After incubation, sections were washed and mounted in Vectashield mounting medium (Vector Laboratories, USA). Immunofluorescence image analysis was performed with the EVOS FL auto cell imaging system (Life technologies, USA), Five random high-powered fields were captured per slide, and the percentage of colocalization was quantified through the mean integrated fluorescence intensity of the Alexa 488-conjugated secondary antibody used to detect the CD4 antigen, and the Alexa 647-conjugated secondary antibody used to detect FOXP3 deposition per treatment group using ImageJ program (7, 44, 46).

### Assessment of Donor–Recipient Functional Microvasculature

The donor–recipient functional microvasculature was examined through whole mount graft fixations (46). Briefly, the transplanted and anesthetized mouse was injected with 100 μl of 1 mg/ml FITC-conjugated *Lycopersicon esculentum* tomato lectin into the inferior vena cava (44). After 5 min of FITC-lectin circulation, the vasculature was flushed with 1% paraformaldehyde (PFA) *via* the aorta for 2 min, and the graft was harvested and fixed in 1% PFA at 4°C for 10 min. Next, the graft was mounted and examined by fluorescence microscopy (EVOS FL auto cell imaging system, Life technologies, USA), and morphometric assessment of % perfused microvasculature was quantified by capturing five random high-powered fields per slide, and the mean integrated fluorescent intensity was calculated per treatment group using ImageJ program (7, 46).

### Assessment of Graft Oxygenation and Microvascular Blood Flow

The level of graft oxygenation (tpO_2_ mmHg) and blood perfusion units (BPUs) during rejection were measured by combined oxygen and blood flow sensors (model NX-BF/OF/E, Oxford Optronix, UK) as described earlier with some modifications (7, 43, 46). Briefly, the transplanted mouse was anesthetized, the graft was exposed, a 23-G needle was used to make a hole in the anterior wall, and a combined oxygen and blood flow sensor was inserted at a <45° angle to contact the epithelium of the opposite wall of the trachea. The sensor was lowered gradually *via* the micromanipulator until the tpO_2_ levels decreases to 5 mmHg or less (indicating a zeroing effect induced by tissue compression). The sensor was then raised in small increments until the tpO_2_ and BPU reading plateaus and a consistent reading was obtained. The consistent pO_2_ was lost as the sensor continues to be lifted off the surface of the airway. The loss of contact of the sensor with the airway epithelium was indicated by a rapid rise to at least 60 mmHg indicating that luminal pO_2_ (rather than tracheal airway tissue pO_2_) was now being sensed by the sensor, and we typically take multiple readings and average the values (43, 48).

### Histopathology and Collagen Staining

To demonstrate subepithelial deposition of collagen, anti-C5a treated and untreated allografts were harvested and processed in Tissue-Tek O.C.T. medium (Sakura Finetek, Japan). A cryostat (HM550; Microm) was utilized to cut 5 µm sections of the tracheal graft and the sections were placed on superfrost/plus slides (Fisher Scientific). Next, slides were stained by H&E and trichrome to detect airway epithelial structures and collagen deposition as described previously (53) and imaged using a Leica light microscope to localize collagen deposition. Morphometric analysis of collagen deposition was quantified by capturing five random high-powered fields per slide, and pixel numbers were converted into micrometers to calculate mean bandwidth (μm^2^) per treatment group using ImageJ program (7, 54).

### Quantitative PCR

RT-PCR analysis of pro-inflammatory, anti-inflammatory, and angiogenic genes was performed with some modifications (55). Briefly, total RNA from tracheal grafts was extracted using RNeasy mini kit 50 (Qiagen Sciences, MD, USA) and quantified using a NanoDrop 1000 spectrophotometer (NanoDrop Technologies, USA). cDNA from each isolated RNA was synthesized with a high capacity cDNA reverse transcription kit (Applied Biosystems) and real-time PCR was performed using gene-specific primers on an AB 7500 Fast Real-Time PCR system in triplicates (Applied Biosystems) using power SYBR Green (Applied Biosystems). Data were analyzed with integrated software, and expression levels were analyzed by the 2^−ΔΔCt^ method after normalization to the housekeeping gene glutaraldehyde dehydrogenase. We selected genes with expected regulatory (IL-10 and TGF-β), Treg induction-specific (IL-5), proangiogenic [vascular endothelial growth factor (VEGF) and ANGPT1], and inflammatory (IL-6) effects. A complete list of individual primers used in the present study is shown in Table 2.

**Table 2:** Sequence of primers for RT-PCR analysis.

Gene	Sequence	Refseqs
IL-5	Forward	TGGGGGTACTGTGGAAATGC
	Reverse	CCACACTTCTCTTTTTGGCGG
TGF-β	Forward	ACTGGAGTTGTACGGCAGTG
	Reverse	GGCTGATCCCGTTGATTTCC
IL-10	Forward	GTAGAAGTGATGCCCCAGGC
	Reverse	GGAGAAATCGATGACAGCGCC
VEGF-a	Forward	TATTCAGCGGACTCACCAGC
	Reverse	CTGGGACCACTTGGCATGG
ANGPT1	Forward	GCTTGGCTTGGATGTGCAAC
	Reverse	TTAGTACCTGGGTCTCAACATCTG
IL-6	Forward	CTCATTCTGCTCTGGAGCCC
	Reverse	TGTGACTCCAGCTTATCTCTTGG

### ELISA

Quantitative estimation of serum cytokine levels was performed by Milliplex MAP Mouse Th17 Magnetic Bead (Cat # MTH17MAG-47K), briefly, blood was collected in BD vacutainers, incubated at room temperature for at least 30 min, and then processed at 1,200 RCF for 10 min, followed by serum aliquots into cryovials and stored at −80°C. As suggested by the manufacturer, samples or standards (6-point dilution) were mixed with antibody-linked magnetic beads on a 96-well plate and incubated overnight at 4°C with shaking. Plates were washed twice with wash buffer in a Biotek ELx405 washer. Following a 1-h incubation at room temperature with biotinylated detection antibodies, streptavidin-PE was added for 30 min with shaking. Plates were washed as above, and PBS added to wells for reading in the Luminex 200 instrument, with a lower limit of 100 beads per sample per cytokine. Each sample was measured in duplicates.

### Statistical Analysis

GraphPad™ Prism software version 5 was used for statistical analysis to compare treated and untreated groups over time. Differences between various groups at multiple time points were compared using two-way analysis of variance (ANOVA) with *post hoc* Bonferroni correction for multiple comparisons while results at one time point were analyzed by one-way ANOVA or two-tailed *t*-test and a *p*-value <0.05 was considered as significant.

## Results

### Blocking C5a is Associated With CD4^+^CD25^+^FOXP3^+^ Treg Increase

Previously, it was reported that pro-inflammatory activities of C5a regulate adaptive immune responses, and affect the development of Tregs through C5aR signaling, which drives Th1 maturation (56–58). Here, we tested the impact of pharmacological C5a blockade by the l-aptamer AON-D21 on the frequency of Tregs in circulation and in grafts during allograft rejection. AON-D21, an improved form of the specific C5a inhibitor AON-D19, had been reported earlier to promote microvascular recovery and to limit airway remodeling during allograft rejection (7) but its effect on cellular immunity, especially on Tregs had not been investigated.

**Figure 1:**
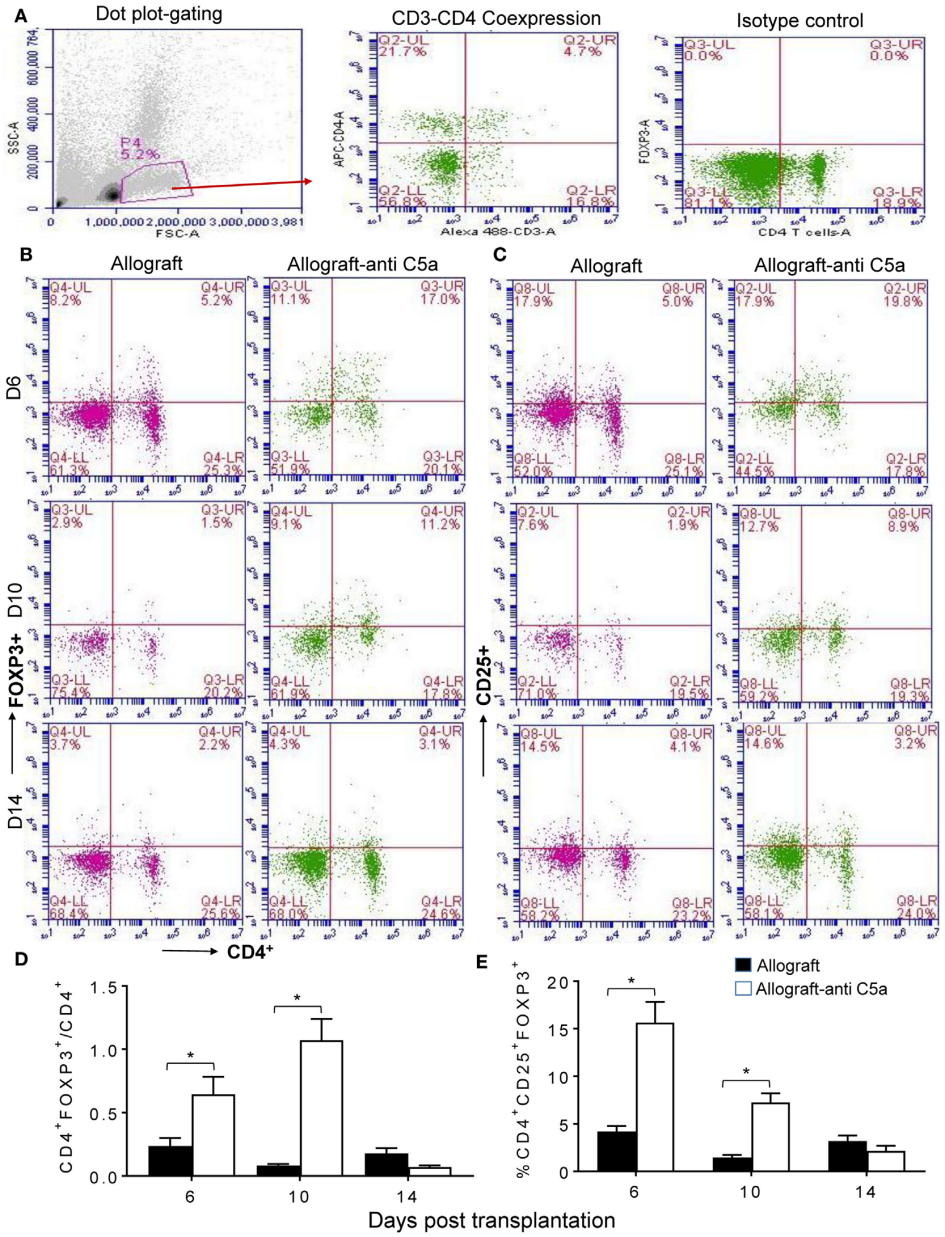
C5a blockade is associated with increased CD4^+^CD25^+^FOXP3^+^ regulatory T cell (Treg) numbers. Flow cytometry analysis of Tregs from peripheral blood of allograft recipients on d6, d10, and d14 posttransplantation: **(A)** Dot plot displaying SSC/FSC of lymphocytes, CD3^+^ positive CD4^+^ T lymphocytes, and isotype control analysis. **(B)** Percentage of CD4^+^FOXP3^+^ lymphocytes in peripheral blood. **(C)** Percentage of CD4^+^CD25^+^ lymphocytes in peripheral blood. **(D)** Ratio of CD4^+^FOXP3^+^ to CD4^+^ lymphocytes. **(E)** Percentage of CD4^+^CD25^+^FOXP3^+^ Tregs of peripheral lymphocytes. Data are presented as means with SE of 4–6 transplants/time point/experiment, and repeats of three different experiments. *p < 0.05.

To assess peripheral and intragraft Treg counts during allograft rejection, we grafted C57BL/6 (B6, H-2^b^) with tracheas from MHC-incompatible BALB/c (H-2^d^) donors. Peripheral CD4^+^CD25^+^FOXP3^+^ Treg counts were determined in blood lymphocytes collected from untreated BALB/c (H-2^d^) → C57BL/6 (H-2^b^) allografts and anti-C5a treated BALB/c (H-2^d^) → C57BL/6 (H-2^b^) allografts at d6, d10, and d14 posttransplantation. Our initial findings showed that C5a neutralization leads to elevated levels of CD4^+^CD25^+^FOXP3^+^ Tregs, compared to untreated control allografts at d6 and d10 posttransplantation (Figures 1A–E). These findings indicate that C5a blockade has potential to modulate ongoing inflammation-mediated injuries and consequently to slow down the process of rejection and microvascular injury. Although there was a significant increase in peripheral CD4^+^CD25^+^FOXP3^+^ Tregs at d6, d10, and d14 posttransplantation under C5a blockade, the CD4^+^FOXP3^+^/CD4 T cell ratio in this group was only on days 6 and 10 but not on day 14 lower than in the control group. This was due to a late drop in CD4^+^FOXP3^+^ lymphocytes while significant increase in CD4^+^ T cells occurred.

**Figure 2:**
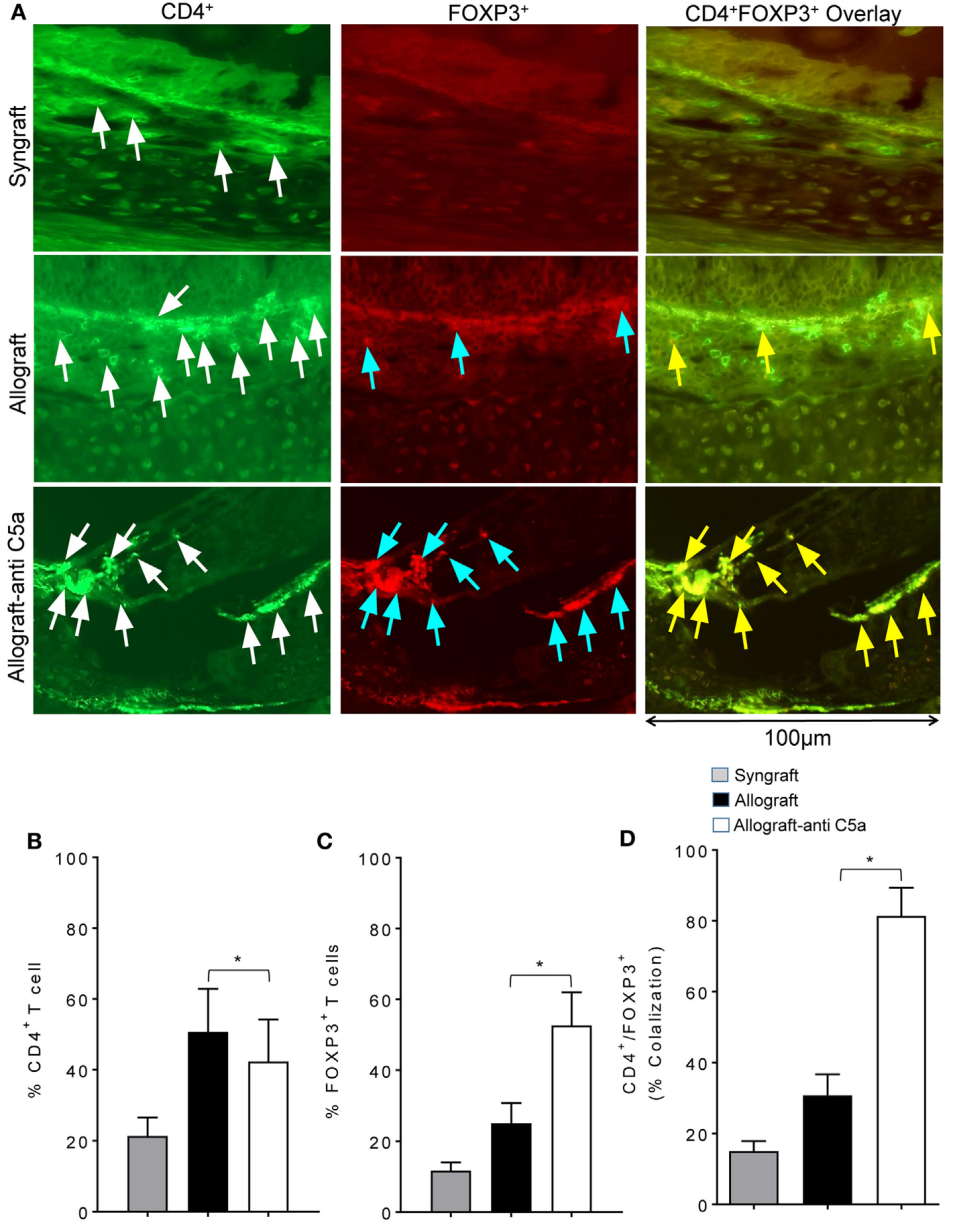
C5a blockade increased infiltration of regulatory T cell (Treg) in allograft. **(A)** Immunofluorescent staining for CD4 and forkhead box P3 (FOXP3) at d10 posttransplantation. **(B–D)** Morphometric analysis of graft infiltrating CD4^+^ T cells, FOXP3^+^ cells, and CD4^+^FOXP3^+^ colocalization (Tregs). Arrows highlighted individual CD4^+^ T cells (white arrows), FOXP3^+^ cells (blue arrows), and CD4^+^FOXP3^+^ Tregs (yellow arrows). Data are presented as means with SE of 3–4 transplants/time point/experiment, and repeats of three different experiments. *p < 0.05. Original magnification, ×40.

Because peripheral CD4^+^CD25^+^FOXP3^+^ Tregs have been associated with improvements in donor–recipient microvascular blood flow, we next investigated whether the observed systemic increase in Tregs is also associated with a surge in subepithelial Tregs or drop in number of CD4^+^ T cells in allograft. We performed immunofluorescent co-staining of anti-C5a-treated allografts for CD4 and FOXP3 at d10 posttransplantation. Immunofluorescence imaging showed an increased subepithelial colocalization of FOXP3 on CD4^+^ T cells in anti-C5a-treated allografts compared to untreated control allografts, which in previous studies overlapped with the day of microvascular rejection in untreated allografts (Figures 2A–D). The systemic increase in CD4^+^CD25^+^FOXP3^+^ Tregs was found to be directly proportional to the increase of intragraft CD4^+^FOXP3^+^ Tregs, which, we strongly believe will take part in maintaining microvascular repair. This latter observation is consistent with the fact that, in the current trachea transplantation model, subepithelial presence of CD4^+^FOXP3^+^ Tregs is associated with microvascular restoration and tissue repair (24, 27).

### Blocking C5a Preserve Donor–Recipient Microvasculature, and Improves Graft Oxygenation and Microvascular Blood Flow

While the effects of C5a inhibition by the related l-aptamer NOX-D19 on vascular reestablishment, tpO_2_ blood perfusion had already been reported earlier (7), here we further delineated the effects of the follow-up drug candidate AON-D21 which surprisingly showed Treg induction in treated allografts. This prompted us to further investigate in detail the effects of C5a blockade on donor–recipient microvasculature, graft oxygenation, and microvascular blood flow. As reported previously, Treg induction has been associated with vital improvements in microvascular blood flow, tpO_2_, and preserved donor–recipient microvascular flow during airway allograft rejection (24, 27). Therefore, the purpose of this study was to further study the effects of pharmacological C5a inhibition on tpO_2_ and blood perfusion during airway allograft rejection. We hypothesized that C5a blockade-mediated Treg induction would reinstate donor–recipient microvasculature, and would thereby ameliorate microvascular flow and tpO_2_. In this therapeutic protocol, anti-C5a treated and untreated allografts were monitored for tpO_2_, microvascular blood perfusion, and occurrence of donor–recipient microvasculature in the course of airway rejection.

**Figure 3:**
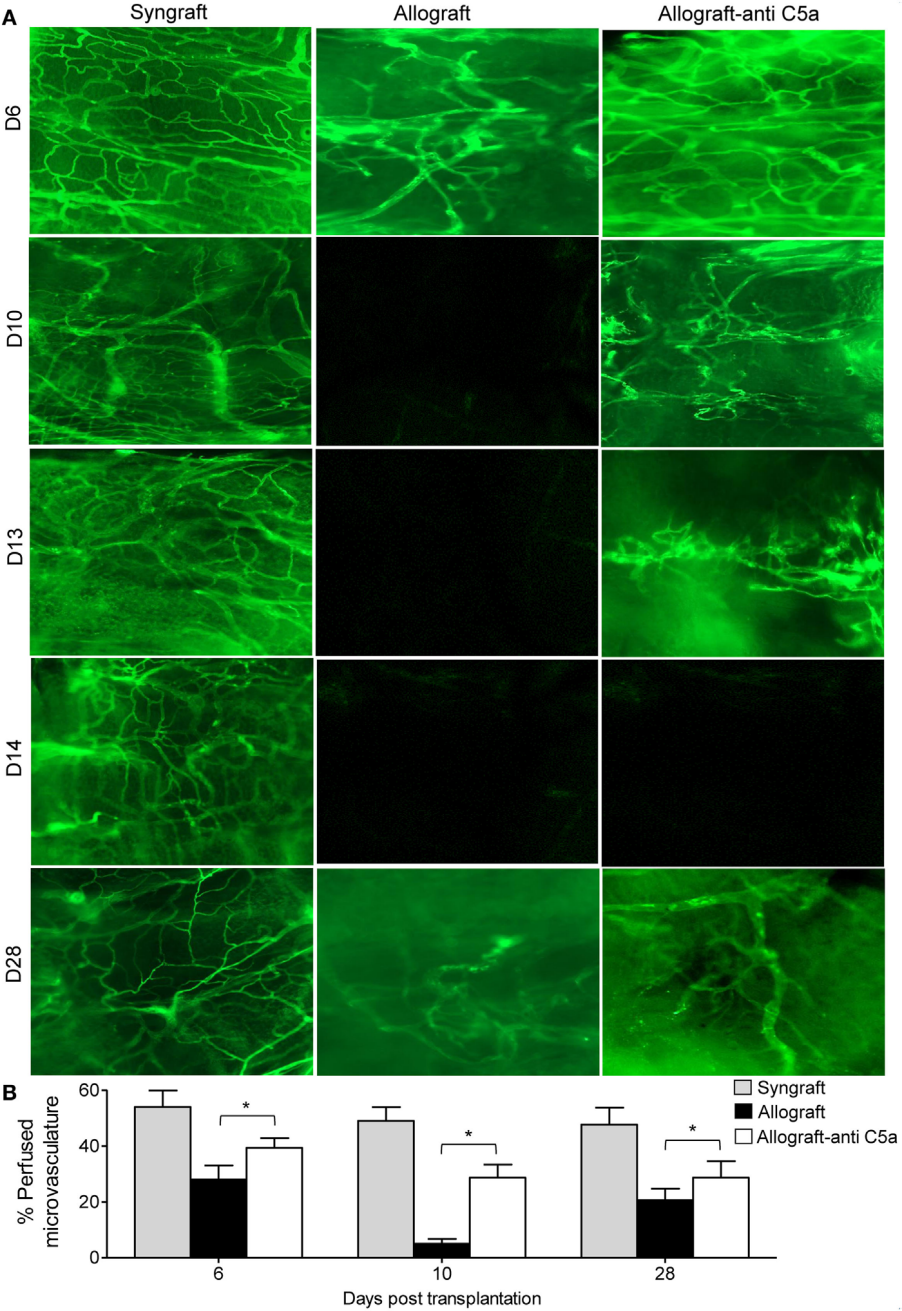
C5a blockade preserves donor–recipient microvasculature. **(A)** Lectin binding assay of whole mount tracheal grafts on d6, d10, d13, d14, and d28. Original magnification, ×20. **(B)** Morphometric assessment of perfused vasculature (lectin-stained vessels/unit area) in allo-transplanted groups at different time points. Data are presented as means with SE of 4–6 transplants/time point/experiment, and repeats of three different experiments. *p < 0.05.

First, to investigate anti-C5a-mediated microvascular reestablishment and associated donor–recipient microvasculature tissue repair in rejecting allografts, we grafted C57BL/6 recipients with tracheas from MHC-incompatible BALB/c donors (allografts) or from C57BL/6 donors (syngrafts). To further investigate microvascular recovery, anti-C5a-treated allografts were examined by FITC-lectin binding assay, which specifically detects the pattern of donor–recipient functional microvasculature during rejection (7, 41, 46). We found that syngrafts remained perfused with high vascular density throughout the period of transplantation. In the allograft setting, anti-C5a-treated allografts showed improved microvascular perfusion at d8, d10–d13, and d28 as compared to untreated control allografts, which lost perfusion and remained poorly perfused with significantly lower vascular density (Figures 3A,B). These findings support the notion that C5a neutralization promotes the microvascular reestablishment. Furthermore, these findings supported that enhanced microvascular density in anti-C5a-treated allografts may be crucial for maintaining high oxygenation and perfused microvascular state.

**Figure 4:**
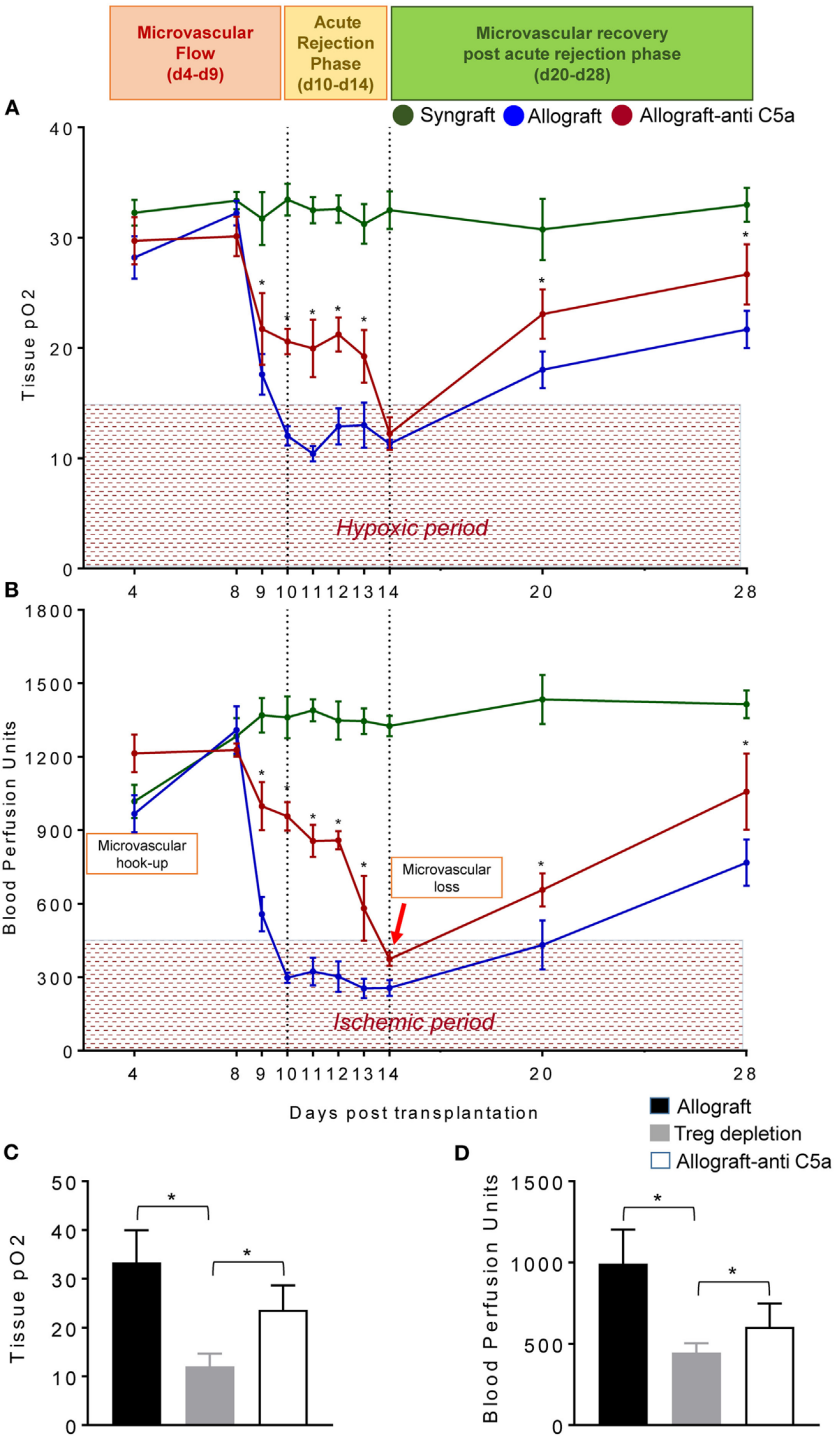
C5a-mediated regulatory T cell (Treg) induction improves tissue pO_2_, blood perfusion. **(A)** Tissue pO_2_ (mean ± SE, mmHg) and **(B)** blood perfusion (mean ± SE, units) were plotted over different time points (d4–d28). **(C,D)** Tissue pO_2_ and blood perfusion were plotted in untreated, Treg depleted, and anti-C5a-treated transplants at d8 posttransplantation. Data are presented as means with SE of 4–6 transplants/time point/experiment, and repeats of three different experiments. *p < 0.05.

To test this, we examined the levels of tpO_2_ and microvascular blood flow (measured in BPUs) in syngrafts, untreated control allografts, and anti-C5a-treated allografts from d4–d28 posttransplantation (Figures 4A,B). Our results demonstrate that BALB/c→C57BL/6 untreated control allografts, BALB/c→C57BL/6 anti-C5a-treated allografts, and C57BL/6→C57BL/6 syngrafts showed a significant increase (*p* < 0.05) in tpO_2_ and BPUs after first graft donor–recipients microvascular hookup at d4, and thereafter all experimental groups remain oxygenated until d8 posttransplantation (Figures 4A,B). At later time points, C57BL/6→C57BL/6 syngrafts remain oxygenated and perfused until d28 posttransplantation without any sign of donor–recipient microvascular loss and associated ischemia. However, BALB/c→C57BL/6 untreated control allografts pass through an extended period of hypoxia and ischemia from d9–d14 and only show a late but inadequate recovery in both tpO_2_ and blood microvascular perfusion by d28 posttransplantation (Figures 4A,B). Next, we examined BALB/c→C57BL/6 anti-C5a-treated allografts, and our initial findings demonstrated significant improvements both in tpO_2_ and blood microvascular perfusion leading to a reduced overall hypoxic and ischemic period of anti-C5a-treated allografts. In detail, our data reveal that anti-C5a-treated allografts remain significantly oxygenated (21.72–19.25 mmHg tpO_2_) and perfused (998-581 BPUs) at d9–d13 and only showed a brief period of hypoxic/ischemic phase around d14 followed by a significant and rapid rise of tpO_2_ and BPUs until d20–d28 posttransplantation (Figures 4A,B). Furthermore, to better understand the mechanism behind the correlation between Tregs, C5a blockade and the microvascular restoration, we first depleted the Tregs with anti-mouse CD25 (clone PC61), and then treated Treg-depleted transplants with the C5a-neutralizing l-aptamer to validate the potential tolerogenic effects of C5a blockade. Our results supported the notion that Treg depletion is associated with a drop in both tpO_2_ and low blood flow state. In addition, subsequent C5a blockade was found sufficient to recover the tissue oxygen and blood flow at d8 posttransplantation (Figures 4C,D).

In summary, syngrafts remain oxygenated/perfused from d4 of microvascular hookup until d28 of posttransplantation without any sign of microvascular loss at d10/d14 but rejecting allografts pass through a comparatively shorter oxygenated phase from d4 to d9 and are progressively hypoxic/ischemic from d10 to d14 posttransplantation, while anti-C5a-treated allografts showed an extended period of graft oxygenation from d4 to d13 followed by a reversible microvascular loss at d14, which confirmed that anti-C5a-treated allografts enjoyed a longer period of oxygenation and blood microvascular flow, and therefore they pass only through a brief phase of hypoxic and ischemic state compared to untreated allografts (Figures 4A,B). This brief phase of low tpO_2_ and BPUs in anti-C5a-treated transplants is followed by a rapid rise in tpO_2_ and BPUs compared to untreated transplants supported the notion that anti-C5a-mediated Treg induction is involved in microvascular improvements (Figures 4A,B). Of note, anti-CD25-mediated Treg depletion subdued tpO_2_ and blood flow, while concomitant C5a blockade improved tissue oxygen and blood flow. Collectively, these findings indicate that C5a blocking delays the onset of acute rejection, and thereby shortens the phase of hypoxia/ischemia as seen in untreated allografts (Figures 4A–D).

### Blocking C5a Limits Airway Epithelial Injury and Prevents Collagen Deposition

**Figure 5:**
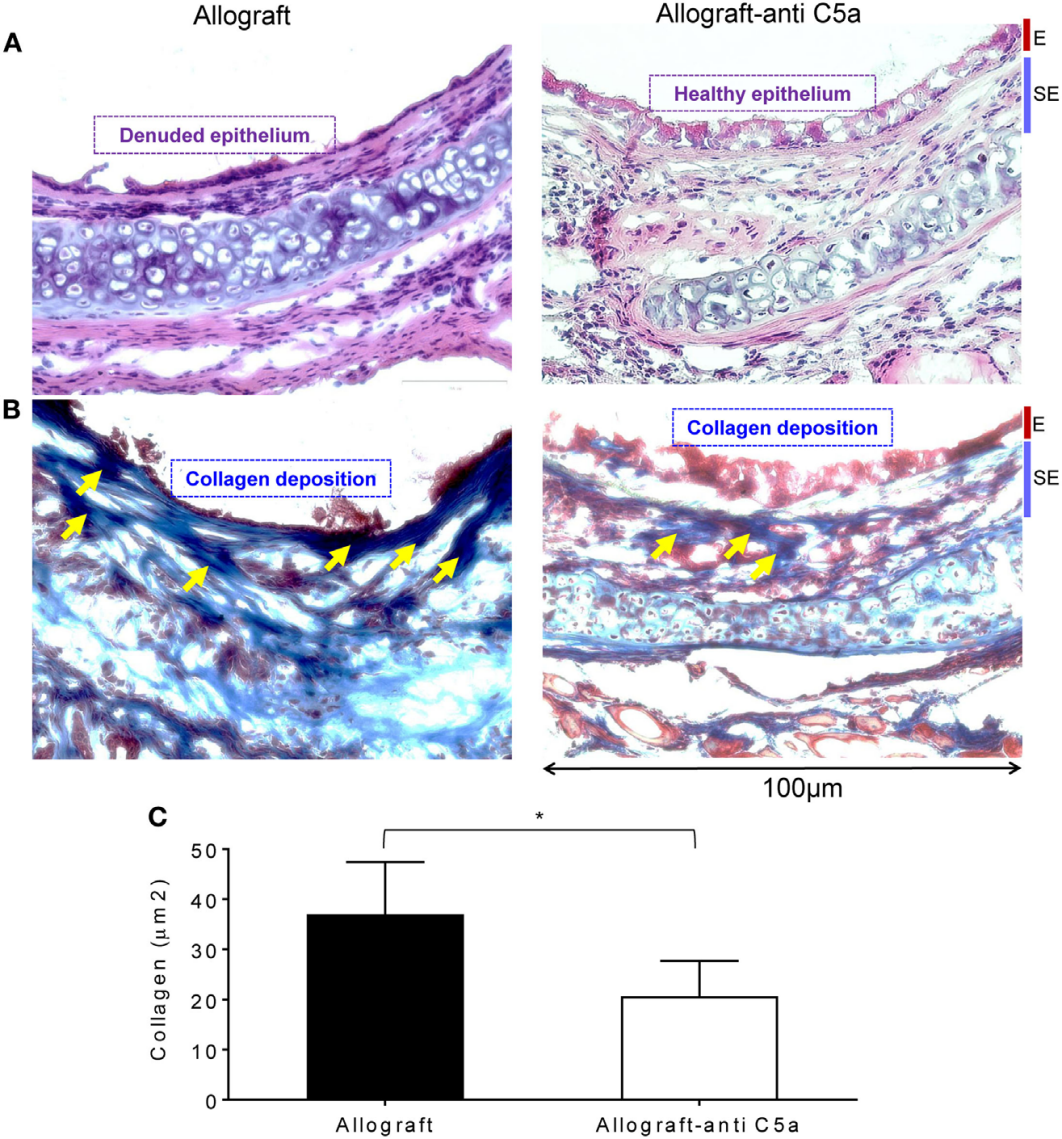
C5a inhibition improves allograft health and prevents collagen deposition. **(A)** H&E staining of BALB/c→C57BL/6 allotransplants on d28 posttransplantation. **(B,C)** Subepithelial deposition and quantification of collagen: blue bands represent subepithelial collagen deposition and quantification of collagen blue bands was performed using ImageJ program. “E” and “SE” designate graft epithelial and subepithelial areas, respectively. Data are shown as means with SE and representative images of at least two different experiments (*n* = 3–4). *p < 0.05. Original magnification, ×40.

Airway epithelial injury and deposition of collagen are the leading pathological events, and key irreversible pathological consequences that proceed when rejecting allografts undergo a state of severe hypoxia/ischemia, and ultimately develop fibrotic remodeling during the terminal phase of CR (46). To investigate these pathological changes, we performed H&E and trichrome staining on untreated allografts and anti-C5a-treated experimental groups on d28 posttransplantation. Microscopic examinations of H&E demonstrated that C5a inhibition resulted in healthier epithelium at d28 as compared to untreated control allografts, which remain denuded and do not show any associated tissue repair/healthy epithelium (Figures 5A). In addition, trichrome staining of anti-C5a-treated allografts showed a significant drop in subepithelial collagen deposition at d28 compared to untreated control allografts (Figures 5B,C).

### Blocking C5a Attenuates Inflammatory Gene Expression While Promotes Regulatory/Proangiogenic Gene Expression

**Figure 6:**
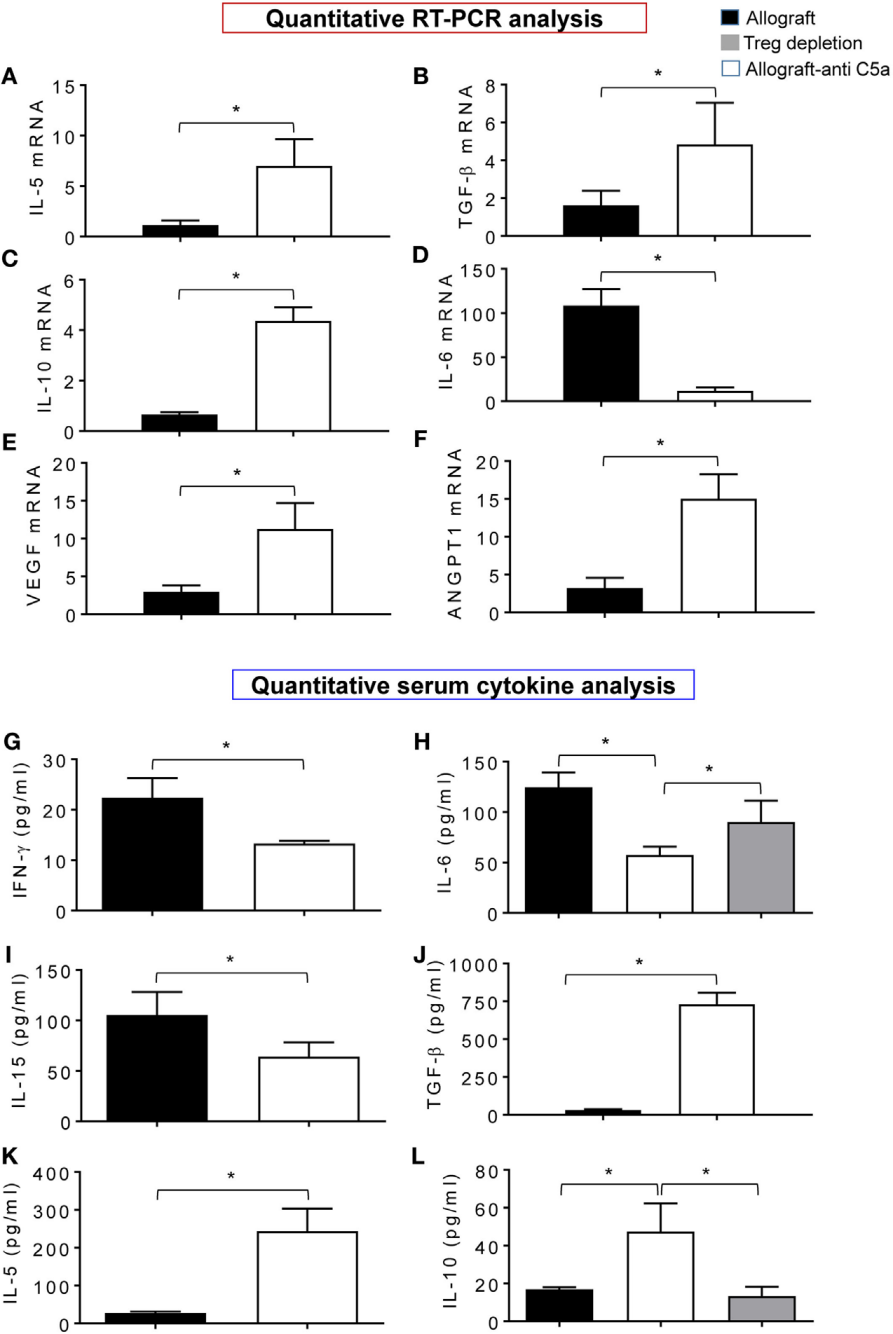
C5a inhibition promotes gene expression and anti-inflammatory cytokine. **(A–F)** Quantitative RT-PCR analysis shows fold change in regulatory and proangiogenic genes IL-5, TGF-β, IL-10, IL-6, vascular endothelial growth factor (VEGF), and AGPT1 mRNA at d10 posttransplantation. **(G–L)** Quantitative analysis of serum cytokines (pg/ml) at d10 posttransplantation. Data are presented as means with SE of 3–4 transplants/time point/experiment, and repeats of three different experiments.

In order to demonstrate that the beneficial effects of C5a neutralization on microvascular-associated allograft improvements, we compared the leukocyte gene expression and serum cytokine levels in treated and control allografts. Quantitative PCR analysis revealed that anti-C5a-treated allografts showed a significant increase in mRNA transcript of IL-5, TGF-β, IL-10, VEGF, and ANGPT1 compared to untreated control allografts (Figures 6A–E). In addition, expression of IL-6, which is responsible for tissue-specific inflammation during acute rejection in untreated control allografts, was reduced in anti-C5a-treated allografts (Figure 6F). Figures 6G–L demonstrated a significant drop in levels of IFN-γ, IL-6, and IL-15, while significant increase in TGF-β, IL-5, and IL-10 serum cytokine levels in anti-C5a treated allografts. Thus, potential effectors of immunomodulatory activity of C5a blockade are IL-10 and IL-5, which most likely affect the microvascular-associated recoveries during transplantation. We next investigated if pharmacologic C5a blockade promotes expression of gene transcripts relevant for allograft rejection, in anti-C5a-treated allografts and untreated control allografts at d10 posttransplantation. These findings indicate that enhanced microvascular reestablishment in anti-C5a-treated allograft recipients occurred mainly because of turning on of proangiogenic mRNA gene transcripts, which facilitated the process of microvascular reestablishment.

Furthermore, to confirm the cellular source of these pro-inflammatory cytokines and regulatory cytokines, we collected serum at d10 from Treg-depleted allografts along with control allografts, and quantified IL-10 (regulatory cytokine) and IL-6 (pro-inflammatory cytokine). Our initial data shows that “Treg-depleted” serum samples were found low in IL-10 levels compared to allografts, while IL-6 levels were not affected compared to allograft controls. These data raise the possibility of Tregs as one of the major source of IL-10 as seen in serum from anti-C5a-treated allografts which had more IL-10 than vehicle-treated allografts (Figures 6H,L).

## Discussion

The association between the complement pathway and Tregs has been well reported (59). Tregs play a crucial role in maintaining immune tolerance in both preclinical studies and are also associated with improved long-term transplant outcomes in clinical studies (60, 61).

Herein, we tested, whether and how pharmacologic C5a inhibition by the C5a-binding aptamer AON-D21 modulated the peripheral and intragraft numbers of Tregs during orthotopic trachea transplantation. We observed that targeted blocking of C5a signaling augments peripheral CD4^+^FOXP3^+^Treg numbers improves microvascular blood flow, and, as a consequence, limits tissue injury and collagen deposition during allograft rejection. The present study was designed to evaluate whether induction of Tregs after complement C5a blockade supports the reestablishment of microvasculature of rejecting allograft and improves tpO_2_ and blood perfusion. Our results indicate that C5a blockade induces an increase in peripheral CD4^+^CD25^+^FOXP3^+^ Tregs and within the graft which is accompanied by an upregulation of IL-5, TGF-β, IL-10, VEGF, and ANGPT1, and reduced IL-6 mRNA gene expression. In additional studies of cytokine concentrations, C5a blockade led to a significant increase in serum levels of TGF-β, IL-5, and IL-10, while reduced levels of IFN-γ, IL-6, and IL-15 were observed. Altogether, this favorable environment seems to contribute to an improved, i.e., anti-inflammatory and more angiogenic balance of the immune system during the allograft microvascular repair, and may help to establish a state of immunotolerance. Earlier, it was demonstrated that blocking/or genetic deficiency of C3aR/C5aR on Tregs enhanced their *in vitro* and *in vivo* suppressive activity and prolonged allogeneic skin graft survival (40). Additional studies demonstrated that C3aR/C5aR deficiency/or blockade stimulates murine Tregs, stabilizes FOXP3 gene expression, prevents Treg conversion to IFN-γ/TNF-α producing T effector cells, and thereby limits graft versus host disease (59,). Liu et al. reported an antagonistic effect between CD4^+^CD25^−^ T cells and CD4^+^CD25^+^ Tregs on macrophage M1 and M2 polarization, respectively (62, 63). In addition, CD25^high^FOXP3^+^ were the dominant cell type which indicated that C5a inhibition supports the induction of this Treg subgroup, which has stronger immune-suppressive properties than CD25^low^FOXP3^+^ Tregs. FOXP3 is the most specific intracellular Treg marker; it is strongly associated with the survival and function of Tregs (64–66). Our results demonstrated that CD4^+^CD25^+^ cells from treated allograft recipient blood express high levels of FOXP3, whereas FOXP3 and CD25 expression in untreated allograft recipients was considerably lower. Our results also demonstrated that reduced infiltration of CD4^+^ effector T cells in anti-C5a-treated allograft are in agreement with previous findings which supports the notion that CD4^+^CD25^+^FOXP3^+^ cells are more suppressive (67). BALB/c→C57BL/6 allografts exhibited low expression of FOXP3 and CD25 during allograft rejection and, as expected, low tpO_2_ and blood perfusion levels were noted. To check the effect of C5a neutralization in BALB/c→C57BL/6 allografts, we used a novel anti-C5a l-aptamer, AON-D21, and showed that targeted blocking resulted in improved microvascular repair, improved tpO_2_ and a higher number of peripheral and allograft resident FOXP3^+^ Tregs. In addition, CD4^+^ T cell-mediated inflammation was limited, suggesting that pharmacologic C5a inhibition triggers the induction of CD25^+^FOXP3^+^ Tregs which support the reparative phase of rejecting allograft through microvascular reestablishment. Furthermore, reestablishment of microvascular flow in C5a-treated allografts is highly correlated with the maintenance of normal airway structural recovery which is characterized by relatively low subepithelial fibrosis and healthy ciliated pseudostratified columnar epithelium compared to severely damaged airway epithelium and significantly higher collagen deposition in untreated allografts. Clinical studies from obstructive bronchitis reported that epithelial cell destruction during persistent inflammation occurs due to a variety of inflammatory mediators in small airways, which impede epithelium regeneration and promote fibro-proliferation due to aberrant tissue repair. These epithelial injuries have been recognized as a key intermediate step that is ultimately leading to obliterative airway disease (68–71). In conclusion, we reported that blockage of C5a triggers activation and expansion of CD4^+^FOXP3^+^ circulating Tregs. The delineation of this formerly uncharacterized mechanism favors the possibility that targeted blocking of C5a could be exploited to rescue microvascular loss during allograft rejection, and limiting C5a/C5aR signaling on Tregs could be used to induce Treg activation and regulatory functions, thereby enhancing Treg responses in allografts. It is known that complement activation and signaling through C3aR and C5aR suppress regulatory functions of murine natural regulatory T cells through FOXP3 expression (11). In addition, immune cell-derived complement also impacts human T-cell immunity and GvHD, highlighting the scope of complement blockade for GvHD treatment (41, 73). These preclinical and clinical findings provide proof of concept that C5a blockage is a key target for facilitating Treg-mediated transplant tolerance (7, 72). Recently, it was reported that anti-C5a treatment is associated with a decrease of pro-inflammatory chemokines, inhibition of infiltration of neutrophils, and enhanced Th2 response in a murine model of colitis (74). C5a receptors also play a key role in modulating alloreactivity of Tregs which are the main regulatory cell responsible for tissue repair during extensive allograft injury, and mediate tolerance to alloantigens in humans (56). Complement antagonism as a therapeutic alternative or addition necessitates a comprehensive information about the mechanisms of complement activation, cell types, and cell mediators responsible for the inflammation and associated tissue injuries. Various approaches to inhibit the complement system have been tested in preclinical models, particularly in ischemia/reperfusion injury, and in transplantation. Most of the previous investigations had proposed that a successful treatment with a specific complement-inhibitor is the ultimate proof that complement plays an essential role in the pathogenesis of disease.

**Figure 7:**
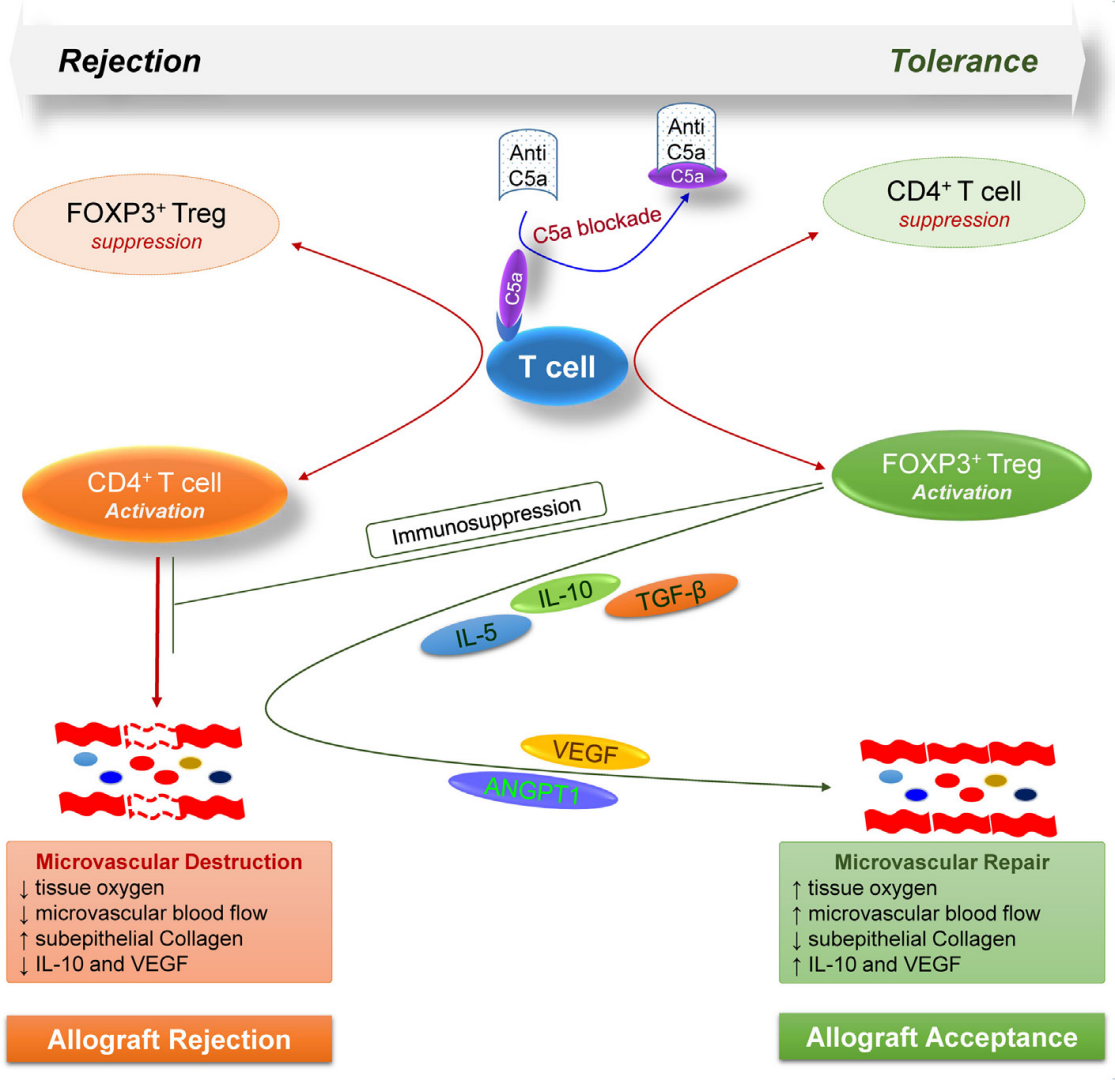
Model illustrates how, during allograft rejection, complement C5a blockade modulates the balance of regulatory and effector T cells, and repairs transplant associated microvascular flow through the release of various regulatory and angiogenic mediators.

Taken together, these findings highlight the key modulatory effects of complement on T cell-mediated alloimmune reactions, and further demonstrate a proof of concept that targeting C5a blockade could facilitate Treg-mediated tolerance to alloantigens (Figure 7). Consequently, the anti-C5a l-aptamer AON-D21 could be exploited to promote induction of immune tolerance to alloantigens in clinical settings as standalone or combination therapy to subdue the severe toxic effects of ongoing immunosuppressive regimens.

## Author Contributions

All major experiments, data analysis, and manuscript writing were performed by MK. AV provided AON-D21 and reviewed the manuscript. FA contributed in immunostaining, flow cytometry, and PCR. HA contributed in histology and immunofluorescence staining experiments, while AA and DB provided key suggestions during the whole study.

## Conflict of Interest Statement

AV is co-inventor of AON-D21 and has co-founded APTARION biotech AG, which holds intellectual property rights on this substance and its use. Author AV is shareholder and employee of Aptarion biotech AG, Germany, which holds intellectual property rights on the C5a-neutralizing L-aptamer used in this study, and is co-inventor of this substance. All authors declare that they have no competing interests as defined by Journal, or other interests that might be perceived to influence the results and discussion reported in this paper.

## Abbreviations

ANOVA, analysis of variance; BPUs, blood perfusion units; d, day; CR, chronic rejection; FOXP3, forkhead box P3; FITC, fluorescein isothiocyanate; AON-D21, anti-C5a L-aptamer; OTT, orthotopic tracheal transplant; PBS, phosphate buffered saline; PFA, paraformaldehyde; SC, Subcutaneous injection; tpO_2_, tissue oxygenation; Tregs, regulatory T cells; VEGF, vascular endothelial growth factor.
